# Profiles of Amino Acids and Acylcarnitines Related with Insecticide Exposure in *Culex quinquefasciatus* (Say)

**DOI:** 10.1371/journal.pone.0169514

**Published:** 2017-01-13

**Authors:** Abdiel Martin-Park, Mayra A. Gomez-Govea, Beatriz Lopez-Monroy, Víctor Manuel Treviño-Alvarado, María del Rosario Torres-Sepúlveda, Graciela Arelí López-Uriarte, Olga Karina Villanueva-Segura, María del Consuelo Ruiz-Herrera, Margarita de la Luz Martinez-Fierro, Ivan Delgado-Enciso, Adriana E. Flores-Suárez, Gregory S. White, Laura E. Martínez de Villarreal, Gustavo Ponce-Garcia, William C. Black, Irám Pablo Rodríguez-Sanchez

**Affiliations:** 1 Department of Microbiology, Immunology, and Pathology, Colorado State University, Fort Collins, Colorado, United States of America; 2 Departamento de Zoología de Invertebrados, Facultad de Ciencias Biológicas Universidad Autónoma de Nuevo León, San Nicolás de los Garza, Nuevo León, México; 3 Cátedra de Bioinformática, Escuela de Medicina, Tecnológico de Monterrey, Monterrey, Nuevo León, México; 4 Departamento de Genética, Facultad de Medicina, Universidad Autónoma de Nuevo León, Monterrey, Nuevo León, México; 5 Unidad Académica de Medicina Humana, Laboratorio de Medicina Molecular Universidad Autónoma de Zacatecas, Zacatecas, Zacatecas, México; 6 Facultad de Medicina, Universidad de Colima, Colima, México; 7 Instituto Estatal de Cáncer, Secretaria de Salud de Colima, Colima, México; 8 The Coachella Valley Mosquito and Vector Control District, Indio, California, United States of America; Universidade Federal de Vicosa, BRAZIL

## Abstract

*Culex quinquefasciatus* Say is a vector of many pathogens of humans, and both domestic and wild animals. Personal protection, reduction of larval habitats, and chemical control are the best ways to reduce mosquito bites and, therefore, the transmission of mosquito-borne pathogens. Currently, to reduce the risk of transmission, the pyrethroids, and other insecticide groups have been extensively used to control both larvae and adult mosquitoes. In this context, amino acids and acylcarnitines have never been associated with insecticide exposure and or insecticide resistance. It has been suggested that changes in acylcarnitines and amino acids profiles could be a powerful diagnostic tool for metabolic alterations. Monitoring these changes could help to better understand the mechanisms involved in insecticide resistance, complementing the strategies for managing this phenomenon in the integrated resistance management. The purpose of the study was to determine the amino acids and acylcarnitines profiles in larvae of *Cx*. *quinquefasciatus* after the exposure to different insecticides. Bioassays were performed on *Cx*. *quinquefasciatus* larvae exposed to the diagnostic doses (DD) of the insecticides chlorpyrifos (0.001 μg/mL), temephos (0.002 μg/mL) and permethrin (0.01 μg/mL). In each sample, we analyzed the profile of 12 amino acids and 31 acylcarnitines by LC-MS/MS. A *t*-test was used to determine statistically significant differences between groups and corrections of q-values. Results indicates three changes, the amino acids arginine (ARG), free carnitine (C0) and acetyl-carnitine (C2) that could be involved in energy production and insecticide detoxification. We confirmed that concentrations of amino acids and acylcarnitines in *Cx*. *quinquefasciatus* vary with respect to different insecticides. The information generated contributes to understand the possible mechanisms and metabolic changes occurring during insecticide exposure.

## Introduction

The *Culex pipiens* complex comprises three main species: *Culex pipiens* Linnaeus, *Culex quinquefasciatus* Say and *Culex pallens* Coquillet [1]. *Cx*. *pipiens* complex are major vectors of lymphatic filariasis caused by *Wuchereria bancrofti* in tropical and subtropical regions of Asia, Africa, Central and South America, and the Pacific Islands. *Cx*. *quinquefasciatus* is also the main vector for arboviral infections, such as West Nile virus, St. Louis encephalitis, Sindbis and Rift Valley fever viruses. Members of the *Cx*. *pipiens* complex demonstrate a significant variation in their host range, feeding behavior and female diapause [2–15]. In *Cx*. *quinquefasciatus* the pyrethroid resistance has been documented [16–24]. Pyrethroid resistance in *Culex* spp are conferred by two major mechanisms: detoxification by enhanced cytochrome P_450_ monooxygenases [25] as well as target site insensitivity (*kdr*) (i.e. an L1014F mutation in the voltage sodium channel *gene*) [26]. Previously, Hardstone [27] selected a permethrin resistant (1.300-fold) strain of *Cx*. *quinquefasciatus* (ISOP450) through backcrossing permethrin-resistant JPAL into an SLAB (susceptible) genetic background. Permethrin resistance in ISOP_450_ is mono factorial and due solely to cytochrome P_450_-mediated detoxification. On the other hand, it has been well documented that deltamethrin is one of the most potent insecticides targeting *Cx*. *quinquefasciatus* [27–30]. Besides, Culex populations may be affected by the extensive and intensive use of insecticides even when these species are not being targeted [31]). In this context, organophosphate (OP) and carbamate (CM) insecticides prevent the hydrolysis through inhibiting the enzyme acetylcholinesterase [32]. The cross-resistance is a problem involved in Culex where cytochrome monooxygenases (P450s) have particular interest as they are critical for the detoxification and/or activation of xenobiotics such as drugs, pesticides, plant toxins, chemical carcinogens and mutagens [33].

Selective pressure from xenobiotics (antibiotics, insecticides, herbicides) is a serious problem threatening the control programs for the organism of medical or economic importance. Furthermore, there are some cases documenting that resistance is increasing in a broad range of target organisms [34, 35]. Insecticide resistance in mosquitoes can be divided into two main mechanisms: (a) the overproduction of detoxifying enzymes that sequester and/or degrade the insecticide before it reaches the nervous system (metabolic detoxification) and (b) mutations in the target site that render them less sensitive to the insecticide [36–38]. In this context, storage proteins constitute a primary source of amino acids and energy for metamorphosis [39], hormones and components of the cuticle and participate in immunity [40, 41].

Previous investigations of mitochondrial physiology in flight muscles from *Ae*. *aegypti* revealed that the mitochondria performs an important role in DDT-sensitive reactions in both respiration and ADP phosphorylation [42].

Acylcarnitines are metabolites of beta oxidation of fats which are good biomarkers for early diagnosis of metabolic disorders in mammals [40–43]. With the introduction of tandem mass spectrometry (MS/MS) in clinical chemistry in the 1990s, it became relatively easy to measure acylcarnitines profiles. In these profiles, the mass-to-charge ratio reflects the length and composition of the acyl chain [44]. Reference ranges of acylcarnitines have been established for obese rats, adult canines, plants, and recently in horses [45–48]. Lipid metabolism pathways have been extensively studied as fats are sources for flight muscle energy in some mosquito species.

For the aforementioned reasons, the understanding of mitochondrial functional processes in insects could have potential implications for dispersal, reproduction, survival, aging, insecticide resistance and pathogen transmission.

To date, the role of aminoacid and acylcarnitines in insecticide resistance remain unknown. Changes in these metabolites may result from altered biochemical pathways due to alterations or disease [49]. Changes in acylcarnitines and amino acid profiles are a powerful diagnostic tool for metabolic alterations [50], and could be useful indicators of deviations in metabolic states. Monitoring these changes could be an opportunity to advance understanding the mechanisms of insecticide resistance, finding new control strategies. The objective of the present study was to measure the amino acid and acylcarnitine concentrations in larvae of *Cx*. *quinquefasciatus* after the exposure to three different insecticides, two organophosphates and one pyrethroid.

## Materials and Methods

### Mosquito sampling

*Culex quinquefasciatus* strain from Nuevo Leon, northeastern Mexico was propagated in plastic containers (30x40cm) with dechlorinated water along with a 50% aqueous solution of powdered liver protein as a food source for the larval stage. They were maintained under laboratory conditions at a temperature of 27 ± 2°C, relative humidity of 75 ± 2% and a LD 12:12 h photoperiod [51], until adult emergence. Individuals of F_1_ generation were used for all the assays.

### Larvae bioassay

*Culex quinquefasciatus* larvae were exposed to standard bioassays using the diagnostic dose (DD) of the technical grade insecticides: chlorpyrifos 99.5% (0.001 μg/mL), temephos 97.5% (0.002 μg/mL) and permethrin 99.5% (0.01 μg/mL) (ChemService, West Chester, PA) [52, 53]. Three replicates of each insecticide were prepared in standard (weight/volume) alcohol solution. Control groups were placed in recipients containing water and 1 mL of alcohol. After 24 h of insecticide exposure all larvae that were found alive were separated from dead ones. The alive larvae were placed individually in 1.5 mL tubes at -80°C for the metabolomics analysis. At the same time and as the same way, unexposed larvae of *Cx*. *quinquefasciatus* were stored.

### Preparation of samples and extraction of metabolites

Three replicates of 10 live larvae were homogenized in 500 μL of ddH_2_O (unexposed and exposed) and placed in 1.5 mL Eppendorf tubes and centrifuged for 10 seconds. The lysate was recovered with a sterile syringe in which was attached one 0.22 uM acrodisc. The filtrate was placed in another tube. Finally, 30 μL from the filtered lysate was added using filter paper (S&S903).

### Metabolomics analysis

Each sample was analyzed for 12 amino acids and 31 acylcarnitines [a 3.2 mm circle was obtained using a Wallac DBS Puncher (PerkinElmer, Waltham, MA, USA)] from the dry filter paper. A NeoBase non-derivatized LC-MS/MS kit (Perkin Elmer) was used to obtain the metabolites of interest, following the manufacturer’s instructions. A solution included in the kit containing internal standards labeled with stable isotopes was used for quantifying the metabolites of interest. The samples were analyzed by LC-MS/MS (API 2000, ABSciex, Framingham, MA, USA) coupled to a micropump and an autosampler (Series 2000, Perkin Elmer). Sample analysis was performed with multiple reaction monitoring using Analyst 1.6.2 Software (ABSciex) and the NeoBase database. The results were interpreted using Analyst 1.6.2 Software.

### Statistical analysis

The statistical analysis was performed using R [54]. To normalize the raw measurements, we set the sum of all values in a sample to 1. This was achieved by dividing each value by the sum of all values per sample. These normalized values were then converted to logarithms to handle extreme values. A *t*- test was used to determine statistically significant differences between groups (p<0.05).

## Results

### Unexposed and exposed larvae to different insecticides

**Fig 1:**
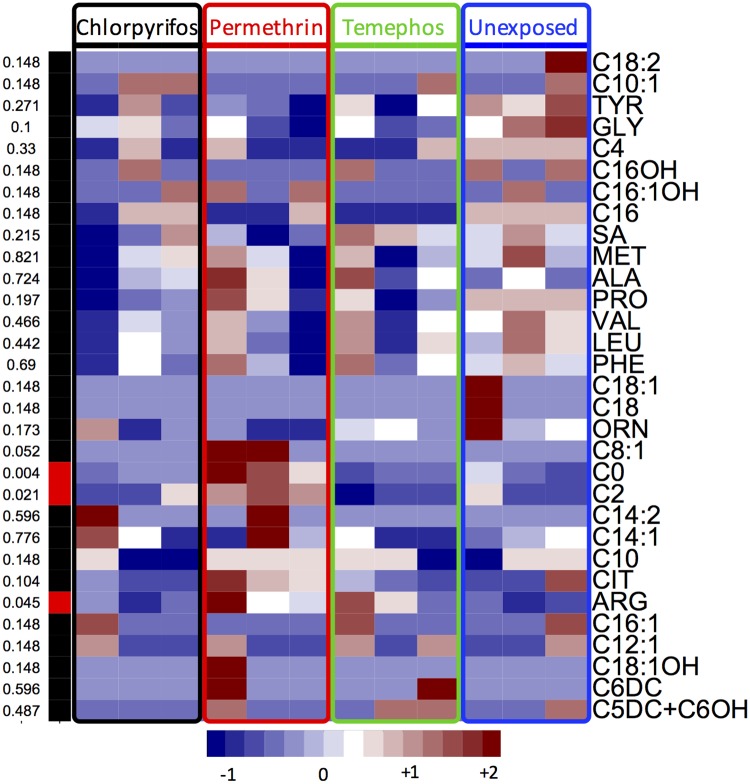
Heat map representing concentration of metabolites analyzed. Columns to the left show the p-value showing statistical significance in red. Insecticides (chlorpyrifos, permethrin and temephos) and the control group (larvae unexposed) are shown in first columns. Acronyms on the right are the metabolites. In the heat map, blue, white, and red, indicates low, median and high concentration, respectively.

A total 43 metabolites (12 aminoacids and 31 acylcarnitines) were determined. From these, 3 metabolites from exposed compared to unexposed larvae to different insecticides (chlorpyrifos, temephos and permethrin) were significantly different. In addition, 34 of these metabolites did not show differences whereas 6 were not detected (Fig 1).

**Fig 2:**
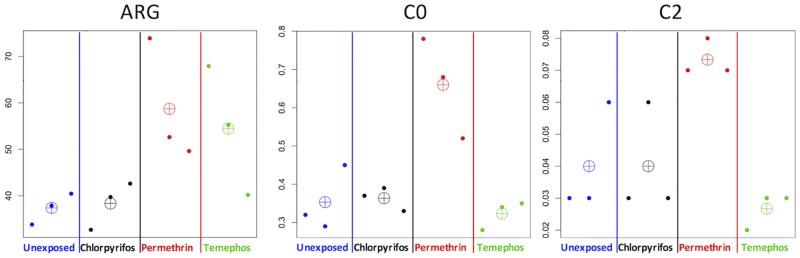
Mean and observed measurements of identified metabolites. Arg, C0, and C2 from left to right. Each dot corresponds to a sample. Crossed circle represent the mean for each treatment group. The vertical axis shows the measurements. The horizontal axis represents samples and treatment groups.

We observed changes in the profile of three metabolites, two of which corresponded to acylcarnitines (C0 and C2) and one, to the amino acid arginine (ARG). The concentration of C0 did not show significant difference in larvae exposed to chlorpyrifos and temephos (+0.028 and -0.085 fold changes, respectively) but an increase in permethrin (+0.868-fold change) was detected compared with unexposed larvae. C2 was increased in the presence of permethrin (+0.833-fold change) and decreased in presence of temephos (-0.333 fold changes respectively), meanwhile in chlorpyrifos was not detected. The concentration of ARG increased in larvae exposed to permethrin (+0.571-fold change) and temephos (+0.457-fold change) but not in those exposed to chlorpyrifos (Table 1 and Fig 2).

**Table 1:** Differences in *Cx*. *quinquefasciatus* larvae exposed to different insecticides relative to unexposed.

Metabolite	μg/mL	Fold change		
	Unexposed	Chlorpyrifos (0.001 μg/mL)	Permethrin (0.002 μg/mL)	Temephos (0.01 μg/mL)
**ARG**	37.370	+0.0258	+0.571 **	+0.457 **
**C0**	0.353	+0.0283	+0.868 **	-0.085
**C2**	0.040	0	+0.833 **	-0.333

** Statistical difference (p<0.05)

## Discussion

Insecticide resistance is a worldwide concern and understanding the resistance mechanisms is essential for vector control strategies. Today pyrethroid insecticides are the most used worldwide and represent approximately 25% of the market for insecticides [55]. In a few years mosquitoes have developed a number of adaptations to insecticides. Many studies are focused in the possible gen modification that occurs during insecticide exposure and only a few researchers looking for new pathway. Little is known of the biochemical processes that may be involved during an exposure to insecticides. Here we reported the levels of amino acids and acylcarnitines in *Cx*. *quinquefasciatus* larvae during and without exposure to insecticide [56].

Over the past several years, dried blood spot (DBS) sampling technique has emerged as a pertinent method in both qualitative and quantitative bioanalysis context. These can be analyzed by modern analytical, immunological, or genomic detection systems. Advantages such as low volume requirement, transportation and storage without special treatment, better analytes stability, enhanced clinical cooperation in clinical trials, and reduced unforeseeable exposure of analysts to biohazards, make it the most appropriate blood sampling technique. In the present study we used this technique with filtered lysates of alcohol solution of larvae for bioanalysis, toxicokinetic (TK) and metabolomic, approach, obtaining reliable results [57].

We compared the normal levels (unexposed larvae) of amino acids and acylcarnitine profile in contrast to levels from mosquitoes exposed to three insecticides (temephos 0.002 μg/mL, chlorpyrifos 0.001μg/mL and permethrin 0.01μg/mL). Our results show metabolic differences correlated with insecticide exposure in *Cx*. *quinquefasciatus* larvae. An increase was detected in the concentration of C0 in larvae exposed to chlopyrifos (+0.0283 folds), permethrin (+0.0868 folds) and decrease with temephos (-0.085 folds). On the other hand, C2 in larvae exposed to chlorpyrifos was not detected. In presence of permethrin C2 increased (+0.833 folds) and larvae exposed to temephos decreased (-0.333 folds). In the present study, the larvae exposed to insecticide showed differences in the concentrations of acylcarnitines (C0 and C2) and arginine level (ARG), this could be due to metabolic resistance because the overproduction of specific enzymes involving depletion in energy stores and the request of energy necessary for biological function [58]. It is known that the deployment of insecticide resistance mechanisms and particularly the overproduction of detoxifying enzymes require a substantial investment of energetic resources. In *Cx*. *pipiens*, for example, certain resistant genotypes can produce up to 50 times more esterases (EST) than their susceptible counterparts [58]. In other insects, these overproduced EST can represent up to 3% of the total body proteins [59]. There are some studies where the up-regulation of proteases led insects to degrade proteins for their re-synthesis into detoxification enzymes as in *M*. *domestica* when it is exposed to fenitrothion, cyfluthrin and resistant to other insecticides [60–62]). It has been suggested that the proteases have important role in the degradation of proteins for biosynthesis of the up-regulated metabolic proteins, particularly P450s and the other proteins/amino acid involved in the regulation of insecticide resistance [63].

In this study two organophosphates insecticides were used (temephos and chlorpyrifos). Organophosphate insecticides target the active site of the enzyme acetyl cholinesterase (AChE) resulting in excess of acetylcholine synapses and nervous system hyperactivity [64]. This modification generates changes in some behavioral fitness related traits. Also in *Drosophila* some studies showed that nervous system hyperactivation results in depletion in fat deposits by increasing the metabolic rate and decrease in peptidoglycan synthesis [65].

In mammals, carnitine is a low molecular-weight compound that has an obligate role in the mitochondrial oxidation of long-chain fatty acids. It has also been found to have neuroprotective effects enhancing energy in the mitochondria, antioxidant activity, stabilize membranes, modulate gene expression and improve cholinergic neurotransmission. Lipids are needed for the synthesis of amino acids as they are an important source of acetyl groups. Lipids are the most affected molecules as a great excess investment in proteins. They are important source of acetyl groups necessary to synthesize constituent amino acids enzymes during the insecticide resistance [66]. Acylcarnitine (C2) is an acetylated form of carnitine; in mammals, it is synthesized naturally in the body and is responsible for the entry of fatty acids into mitochondria and participates in the cycle of producing carnitine Acetyl-CoA, an essential component of mitochondrial respiration with subsequent energy generation. An increase of C2 is a result of an increase of β-oxidation as a consequence of fitness cost [67]. Changes in individual acylcarnitines may imply changes in specific metabolic pathways like physiological and genetic mechanisms as mitochondrial biogenesis, the antioxidant power, and stabilization of membranes among others [68]. Acylcarnitine profiles should lead to better understanding the mechanism of insecticide resistance. A semi-essential amino acid that plays a significant role in the metabolic processes of ornithine and urea cycles used for the elimination of amino compounds is arginine [69]. Lately, considerable numbers of ARG utilization pathways have been discovered that highlight the importance of ARG as well as an energy source [70,71]. Our results show that the larvae exposed to all insecticides increased levels of this amino acid. These findings suggest an overload of these cycles due to metabolic detoxification [72].

We observed high variance in the measured metabolites (Figs 1 and 2). For instance, ARG was increased in exposure to Permethrin and Temephos except in one out of six larvae, and one control larvae showed slightly lower levels of C2. As most of the larvae die during the insecticide exposure, the observed variation may correspond to diverse mechanisms of resistance that could be reflected in the pattern of amino acid content. The significant findings (Fig 2), in average, changed more consistently across these possible mechanisms and therefore may represent a convergent property of resistance. In this context, further experiments should focus on a higher number of larvae to classify the diverse patterns of resistance which would help to study specific resistance mechanisms.

## Conclusions

The metabolic levels from *Cx*. *quinquefasciatus* larvae exposed to three different insecticides were established. We suggest that sequencing the promoters of metabolic genes involved in insecticide resistance should be the next step. This is the first report of amino acids and acylcarnitines from a non-mammalian organism. The establishment of these values becomes an essential reference in the new age of causative energetic cost in the insecticide resistance. These results could help to a better understanding of the mechanisms of insecticide resistance and can be used to define the metabolic status of field populations. This information will help for future studies in where these parameters will be involved in indirect toxic exposures and its influence on insecticide resistance in mosquitoes.

## Supporting Information

S1 Table Complementary Table: Concentration of acylcarnitines and amino acids in *Cx*. *quinquefasciatus* larvae unexposed in ascendent concentrations. (DOCX)
