# Supplementary material for: Profiles of Amino Acids and Acylcarnitines Related with Insecticide Exposure in Culex quinquefasciatus (Say)
Source: PLoS One. 2017 Jan 13;12(1):e0169514. doi: 10.1371/journal.pone.0169514 (PMC5234828; doi:10.1371/journal.pone.0169514)
Supplement: S1 Table — (DOCX) [file pone.0169514.s001.docx]

**Supporting Information**

**Table S1**. Complementary Table: Concentration of acylcarnitines and amino acids in *Cx*. *quinquefasciatus* larvae unexposed in ascendent concentrations.

| **Acylcarnitines** | | **Aminoacid** | | |
| --- | --- | --- | --- | --- |
| **Metabolite** | **Value** | **Metabolite** | | **Value** |
| C14OH | 0 | SA | 0.51 | |
| C18:1OH | 0 | CIT | 0.61 | |
| C18OH | 0 | TYR | 2.187 | |
| C5 | 0 | ORN | 3.193 | |
| C5:1 | 0 | MET | 5.083 | |
| C6 | 0 | PHE | 11.023 | |
| C10:1 | 0.003 | PRO | 14.29 | |
| C12:1 | 0.003 | VAL | 15.247 | |
| C16:1 | 0.003 | GLY | 23.763 | |
| C16:1OH | 0.003 | LEU | 27.947 | |
| C18 | 0.003 | ARG | 37.373 | |
| C18:1 | 0.003 | ALA | 38.697 | |
| C18:2 | 0.003 |  | |  |
| C10 | 0.007 |  | |  |
| C16OH | 0.007 |  | |  |
| C10:2 | 0.01 |  | |  |
| C12 | 0.01 |  | |  |
| C14 | 0.01 |  | |  |
| C14:2 | 0.01 |  | |  |
| C16 | 0.01 |  | |  |
| C3 | 0.01 |  | |  |
| C3DC+C4OH | 0.01 |  | |  |
| C4DC+C5OH | 0.01 |  | |  |
| C8 | 0.01 |  | |  |
| C8:1 | 0.01 |  | |  |
| C5DC+C6OH | 0.013 |  | |  |
| C6DC | 0.02 |  | |  |
| C14:1 | 0.04 |  | |  |
| C2 | 0.04 |  | |  |
| C4 | 0.05 |  | |  |
| C0 | 0.353 |  | |  |
